# The evolution of quantitative sensitivity

**DOI:** 10.1098/rstb.2020.0529

**Published:** 2022-02-14

**Authors:** Margaret A. H. Bryer, Sarah E. Koopman, Jessica F. Cantlon, Steven T. Piantadosi, Evan L. MacLean, Joseph M. Baker, Michael J. Beran, Sarah M. Jones, Kerry E. Jordan, Salif Mahamane, Andreas Nieder, Bonnie M. Perdue, Friederike Range, Jeffrey R. Stevens, Masaki Tomonaga, Dorottya J. Ujfalussy, Jennifer Vonk

**Affiliations:** ^1^ Department of Psychology, Carnegie Mellon University, Pittsburgh, PA 15213, USA; ^2^ Department of Psychology, University of California-Berkeley, Berkeley, CA 94720, USA; ^3^ School of Psychology and Neuroscience, University of St. Andrews, St Andrews KY16 9AJ, UK; ^4^ School of Anthropology, University of Arizona, Tucson, AZ 85719, USA; ^5^ College of Veterinary Medicine, University of Arizona, Tucson, AZ 85719, USA; ^6^ Center for Interdisciplinary Brain Sciences Research, Division of Brain Sciences, Department of Psychiatry and Behavioral Sciences, School of Medicine, Stanford University, Stanford, CA 94305, USA; ^7^ Department of Psychology and Language Research Center, Georgia State University, Atlanta, GA 30302, USA; ^8^ Psychology Program, Berea College, Berea, KY 40403, USA; ^9^ Department of Psychology, Utah State University, Logan, UT 84322, USA; ^10^ Behavioral and Social Sciences Department, Western Colorado University, Gunnison, CO 81231, USA; ^11^ Animal Physiology Unit, Institute of Neurobiology, University of Tübingen, Tübingen 72076, Germany; ^12^ Department of Psychology, Agnes Scott College, Decatur, GA 30030, USA; ^13^ Domestication Lab, Konrad Lorenz Institute of Ethology, Department of Interdisciplinary Life Sciences, University of Veterinary Medicine Vienna, Savoyenstrasse 1a, Vienna 1160, Austria; ^14^ Department of Psychology and Center for Brain, Biology and Behavior, University of Nebraska-Lincoln, Lincoln, NE 68588, USA; ^15^ Inuyama, Aichi 484-0000, Japan; ^16^ MTA-ELTE Comparative Ethology Research Group, Eötvös Loránd University of Sciences (ELTE), Budapest 1117, Hungary; ^17^ Department of Ethology, Eötvös Loránd University of Sciences (ELTE), Budapest 1117, Hungary; ^18^ Department of Psychology, Oakland University, Rochester, MI 48309, USA

**Keywords:** brain evolution, Weber fraction, quantity discrimination

## Abstract

The ability to represent approximate quantities appears to be phylogenetically widespread, but the selective pressures and proximate mechanisms favouring this ability remain unknown. We analysed quantity discrimination data from 672 subjects across 33 bird and mammal species, using a novel Bayesian model that combined phylogenetic regression with a model of number psychophysics and random effect components. This allowed us to combine data from 49 studies and calculate the Weber fraction (a measure of quantity representation precision) for each species. We then examined which cognitive, socioecological and biological factors were related to variance in Weber fraction. We found contributions of phylogeny to quantity discrimination performance across taxa. Of the neural, socioecological and general cognitive factors we tested, cortical neuron density and domain-general cognition were the strongest predictors of Weber fraction, controlling for phylogeny. Our study is a new demonstration of evolutionary constraints on cognition, as well as of a relation between species-specific neuron density and a particular cognitive ability.

This article is part of the theme issue ‘Systems neuroscience through the lens of evolutionary theory’.

## 1. Introduction

Quantitative sensitivity is an aspect of cognition that is ubiquitous among many species, and many researchers debate the nature of its evolutionary basis across taxa, including in humans and other primates [1–6]. Baboons use numerical estimation to guide troop movement [7,8], desert ants and fiddler crabs navigate by keeping track of the number of steps they have taken [9,10], and social species like hyenas and lions vocalize or approach other conspecific groups only when their group has a numerical advantage [11–17]. A diverse range of animals—from primates to reptiles, fish and insects—can discriminate numerical quantities in laboratory tasks, for example, comparing computerized arrays or sequences of pure tones to peck, touch or approach the numerically larger set [2,4,18–22]. Moreover, animals represent numerical values cross-modally [23–27] and under conditions where dimensions such as area, density and duration are equated, uncorrelated with numerical value or otherwise controlled [2,4,20,28–30].

Behaviour in quantitative tasks is well predicted by the general psychophysical principle Weber's law: discrimination accuracy between the magnitudes of two stimuli is determined by their proportional difference [31,32]. Weber's law reliably predicts performance on nonsymbolic numerical tasks across species [7,20,29,33], through human development [34–36], and across cultures [37]. Weber fraction (*w*) is a measure of quantitative precision that represents the proportion difference between quantities that is needed to reliably discriminate them [36,38,39]. In an investigation of the basis of quantitative decision-making by wild baboons, number better predicted decisions than mass, with wild baboons exhibiting Weber fractions similar to those found for baboons in laboratory settings [7]. Insect number sense has been explored [40], especially in honeybees [41–44], with mixed results on relative versus absolute quantity discrimination that suggest different mechanisms may be at play than in mammals and birds. Bees do not show particularly high quantitative sensitivity; for example, with appetitive conditioning only, bees discriminated between 4 and 8 almost at chance level (54%), while appetitive and aversive conditioning improved discrimination only slightly at this 2 : 1 discrimination ratio (64%) [45].

The ubiquity of quantitative representation, and specifically numerical representation, suggests that number is useful in many animals' natural lives, but its phylogenetic basis is unclear. There are no prior studies that examine quantitative cognition in an explicitly phylogenetic framework across a broad range of species. Investigating species differences has long been one goal of comparative psychology, with integration of principles of evolutionary biology [46–48]. However, modern methods of phylogenetic regression across taxa are novel in the context of cognitive studies [49,50] and can provide insight into the phylogenetic signal or ancestral state of a cognitive trait, as well as if social, ecological or anatomical variables affect cognition when phylogeny is taken into account [48].

Here we develop a computational model of the phylogenetic distribution of quantitative sensitivity, and we examine potential causes of changes in quantitative sensitivity including brain morphology, ecology and domain-general cognition. This model is innovative in that it combines a phylogenetic regression—to control for relatedness between species—with a likelihood model based on the psychophysics of number. Specifically, we assume that performance on number tasks is derived from a linear internal mental scale with linearly increasing Gaussian noise. We model variation in species, individual animal and task, and combine these with a predictor. We model phylogenetic relatedness as a correlation matrix among species effects, where a single parameter determines the degree to which species effects are determined by phylogeny. Together in a Bayesian data analysis, this allows us to test the likely influence of a single factor (e.g. an aspect of brain morphology or ecology), while controlling these other components. Thus, likely underlying causal factors can be inferred by exploring the phylogenetic variation in numerical ability across species and investigating which physiological and ecological factors best predict numerical competence—an approach used previously to investigate the evolution of self-control [49], domain-general cognitive ability [51], visual perspective taking [52], prosocial preferences [53], cultural intelligence [54] and temporal decision-making [55]. Identification of these factors may reveal the selection pressures that have shaped numerical understanding throughout evolution. The potential factors we investigate include brain size and neuronal number and density, socioecology and domain-general cognitive ability, detailed below.

### (a) Features of the brain

One potential factor influencing the precision of quantitative representations is brain size, as it has been proposed that humans’ exceptional cognitive abilities arise from our large brains [56–58]. Larger brains, despite their greater metabolic cost [59], provide the neural tissue necessary to solve more complex problems that an animal may encounter in its environment. Indeed, prior research has demonstrated a relation between some cognitive abilities and measures of brain size. Self-control and general cognitive ability (an aggregate score of invisible displacement, reversal learning, delayed response and other cognitive abilities) have been found to be strongly related to absolute brain size, supporting a hypothesis of cortical reorganization driving the evolution of cognitive abilities [49,51]. When brains increase in size, cortical reorganization becomes necessary as neurons in different parts of the brain are pushed apart. This reorganization can result in larger brains showing more modular organization than smaller brains [60,61]. Measures of relative brain size, which take body size into account, cannot capture the degree of cortical reorganization that may have occurred due to increases in absolute brain size. On the other hand, bigger bodies require more sensory and motor brain tissue; relative brain size accounts for body size and is thus a measure of brain size above and beyond that needed for sensory and motor control. Social learning, behavioural flexibility, tool use, innovation and problem-solving ability have been found to be correlated with relative brain size, supporting a hypothesis that encephalization (an increase in cortex size relative to body size) drives cognitive evolution [62–66]. Though the avian brain would appear to be a special case because it does not have a cortex, the nidopallium caudolaterale (NCL) has been identified as functionally equivalent to the mammalian prefrontal cortex [67,68].

Criticisms of using brain size measurements in comparative biology include that these measures do not actually capture variation in brain size across taxa well [69,70]. Some researchers contend that the number of cortical neurons, rather than brain size, is a better proxy for cognitive ability [71]. Recent advancements have made the quantification of neurons more accurate and efficient [72,73], leading to the discovery, spearheaded by Herculano-Houzel, that number of neurons does not scale with brain size the same way in different taxa [74–81]. For example, as brain size increases, the number of neurons in the primate brain increases more than the number of neurons in the rodent brain, meaning that similarly sized primate and rodent brains contain different numbers of neurons [82–84]. The number or density of cortical/pallium neurons may, therefore, be a more accurate proxy for quantitative ability than brain volume, particularly when comparing species across a range of taxonomic groups.

Ratio-dependent neural responses to numerical stimuli have been demonstrated in the NCL of corvids [33,85], comparable to those found in the intraparietal sulcus area of the cortex in humans [86,87] and nonhuman primates [27]. Given the vast differences in avian and mammalian brain structure, it may also be fruitful to explore other avian brain regions with a comparatively high neuronal density as potential proxies for cognitive ability. Cerebellar neuronal scaling rules also differ across taxonomic groups [84], with songbird and parrot cerebellum neuronal densities (as well as pallium neuronal densities) being higher than those of mammals [79]. Mammals from four orders showed the same coordinated scaling of neuron number in both the cerebral and cerebellar cortices, supporting arguments for integrated functions and selective pressures [88]. Indeed, some researchers argue that the cerebellum has been neglected in studies of vertebrate brain evolution and cognition [89–91].

### (b) Socioecology

Socioecological selective pressures could also influence quantitative ability directly since both dietary and social complexity have been previously linked to differences in cognition. Diet is related to spatial memory in seed-caching birds [92–94], Central and South American primates [95] and lemurs [96]. Bonobos, whose food source is more uniformly distributed in their habitat and who arguably rely on a relatively consistent food source, are more risk averse than chimpanzees, whose food source is more patchily distributed [97]. This and other differences in ecology between bonobos and chimpanzees [98,99] have implications for the cognitive profile of the last common ancestor between humans and *Pan* [100]. More broadly, the foraging required to maintain a particular diet has been hypothesized as one of the driving forces in the evolution of primate cognition [101]. Successful foragers of diverse taxa accurately decide when to keep foraging in the same area and when to move to a new area [102]. This likely requires a finely tuned representation of quantity and the ability to accurately compare multiple quantities. Therefore, dietary factors such as the percentage of fruit in the diet, which reflects the diversity of foraging done by a species, may be related to the precision of species' quantitative representations. A larger home range size and/or a longer day journey length involves more complex navigation of heterogeneous environments on a larger scale and may require more quantitative sensitivity in making ecological decisions with high cost or high gain outcomes in terms of energy acquisition or expenditure.

Navigating complex social systems influences multiple components of cognition in social vertebrate taxa. The social intelligence (or social brain) hypothesis suggests that social complexity drove brain evolution, especially in primates [103–105]. Survival in large social groups requires the ability to keep track of multiple individuals and their relationships, as well as judge whether to engage with another group or not. When baboons disagree about the direction of troop movement, undecided baboons more often choose to go in the direction with more supporters [8], behaviour which is driven by approximate number representations [7]. Additionally, in species that engage in reciprocal altruism, having precise quantity representations could help them keep track of the quantities being exchanged and thus avoid cheaters [106]. In intergroup encounters, assessment of the number of opponents is advantageous across multiple taxa [12,15,16]. Within-group social decision-making, in the context of navigation, predator avoidance, cooperative hunting and mating behaviours also involves quantitative processing [5]. Being able to make accurate quantity judgements, due to a well-tuned quantity representation system, would likely be adaptive for a highly social species. Social group factors like group size could, therefore, be related to numerical precision.

### (c) Domain-general cognitive ability

Finally, domain-general cognitive abilities could influence performance on the quantity discrimination tasks used to measure numerical precision. These tasks could place demands on executive functions like attention, memory and self-control that, while distinct from the number sense itself, influence an animal's ability to discriminate between quantities. For example, species with low self-control could perform poorly on quantity discrimination tasks because they are less able to inhibit incorrect responses.

Our goal was to evaluate these factors quantitatively in mammals and birds (*N* = 33 species) using a novel modelling approach that integrates Bayesian, phylogenetic and number psychophysics components. Specifically, the model jointly fits effects of predictors across species on Weber fraction (*w*), as well as the influence of phylogenetic relatedness, while controlling for species, individual, study and task effects. With the wealth of quantitative discrimination studies across taxa, we implemented this model in the context of a meta-analysis.

## 2. Methods

### (a) Animal quantity discrimination data compilation

To find studies from which Weber fractions could be calculated, Google Scholar literature searches were conducted of ‘animal’ and ‘quantity discrimination’ in March 2015 and May 2017. Studies were included in these analyses if they used one of these three paradigms: match-to-sample, go/no-go or ordinal tasks with visual array stimuli (controlled array paradigm); food stimuli tasks with sequential presentation of food (sequential paradigm); and food stimuli tasks with simultaneous presentation of food (simultaneous paradigm). Data were obtained either from the primary literature or, when possible, directly from the author(s). In total, data were obtained from 49 studies for 33 species and 672 subjects. See electronic supplementary material, table S1 for a complete list of the studies included in these analyses.

Number representation precision was quantified by the Weber fraction (*w*), a value that quantifies the representational precision, assuming the underlying approximate number system is formalized as Gaussian distributions along a mental number line [107]. Smaller Weber fractions correspond to narrower Gaussian curves and more precise quantity representations, while larger Weber fractions reflect increased noise and overlap between Gaussian curves of numbers being compared and poorer quantity discrimination. In our model, Weber fraction was jointly estimated with other parameter effects (species, study, subject, task) per species in a Bayesian model implemented in the Stan programming language (see details under Bayesian analyses section).

### (b) Phylogenetic tree construction

Phylogenetic trees were generated in R using the ape package [108], based on information from The Timetree of Life [109,110], incorporating a 3 Mya divergence of the North Island robin and South Island robin [111], and a 15 000-year-ago divergence of dogs and wolves [112].

### (c) Brain and socioecological data

Most endocranial volume (ECV) and socioecological variable data were acquired from a previous large-scale comparative study [49] (see electronic supplementary material, table S1 for primary sources). Additional ECV and socioecological data were obtained from the literature. When ECV could not be found, it was calculated from brain mass based on brain tissue density of 1.036 g cm^−3^ [113]. Residual brain volume (RBV) is the residuals from a phylogenetic regression of ECV on body mass [49]. Actual cortical/pallium neuron data and cerebellum neuron data for 12 species were obtained from the literature (see electronic supplementary material, table S1 for primary sources). For cerebellum neuron number, an outlier, the African elephant, is included, as models run with the African elephant excluded for cerebellum neuron number and density did not show a major shift in effect (see electronic supplementary material, figure S2 for scatterplot of *w* by cerebellum neuron number with and without the elephant outlier). For the primate species without cortical neuron data, the number of cortical neurons was calculated from brain mass using the following scaling rule: no. of cortex neurons = 37 813 551.018 × brain mass^0.891^ [114]. For the primate species without cerebellum neuron number data, the number of cerebellar neurons was calculated from brain mass using the following scaling rule: no. of cerebellum neurons = 69 640 042.656 × brain mass^0.936^ [114]. Calculating cortical or cerebellar neuronal *density* values for species with missing values was less straightforward because neuronal density and neuronal number are decoupled in primates, i.e. unlike in nonprimate mammals, more neurons does *not* mean larger neurons (and lower density) in primates [75,115]. Therefore, our sample size for neuronal density is smaller than that for neuronal number.

Socioecological variable data, specifically group size, percentage fruit in diet, home range and day journey length, were compiled from the literature (see electronic supplementary material, table S1). Group size was compiled across all taxa, while percentage fruit in the diet, home range and day journey length were compiled for primate species only. Finding comparable social and ecological variable data across primate, nonprimate mammal and avian taxa is not straightforward: for example, finding values for percentage frugivory for corvids is complicated by lack of reliance on fruit and corvid diverse diets with variable and sometimes qualitative mention of fruit-eating [116,117]. For bird taxa generally, other social and ecological variables not applicable to mammals may be more relevant selective pressures; for example, degree of food caching.

Some of the variables were transformed in order to meet the assumptions of the statistical analyses performed: ECV, group size, home range, day journey, cortical neuron number, cerebellar neuron number, cortical neuron density and cerebellar neuron density were log-transformed.

### (d) Domain-general cognitive ability data

Self-control data were obtained for 17 species from a large-scale comparative study (species score was composite score calculated from performance on A-not-B task and cylinder task) [49] and for two species from studies of corvids [118] and parrots [119] (species score was performance on cylinder task). Domain-general cognitive ability data were obtained for 10 primate genera from a meta-analysis [120]. In the meta-analysis study [120], a high general cognitive ability score indicated poor performance, so we reversed the scores in our analyses for clarity (so that high score indicated good performance).

### (e) Bayesian analyses

**Figure 1.**
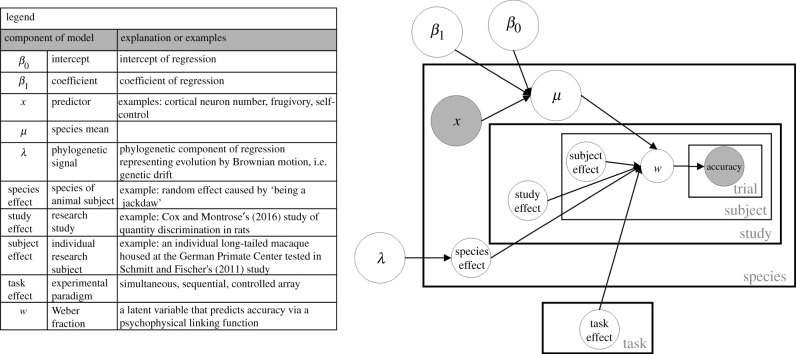
Graphical diagram of the Bayesian model implemented in this study. The model jointly fits effects of predictors (*x*) across species on *w*, as well as the influence of phylogenetic relatedness (lambda, *λ*), while controlling for species, subject, study and task effects. The inferred Weber fractions are converted into a binary prediction by following the common linear-scale-variable psychophysical model that predicts binary accuracy judgements on specific numerical comparisons, which are the observed data for the model.

We used a Bayesian model that combines a phylogenetic regression with a likelihood that is grounded in number psychophysics. Specifically, the regression assumes that there was a linear effect of each single predictor (outlined above) on the mean log Weber fraction for a species. The phylogenetic component includes a correlation that derives from the phylogenetic tree, but the strength of this correlation is determined by a free parameter, lambda (*λ*) [48,121–123]. Finally, the inferred Weber fractions *w* are converted into a binary prediction by following the common linear-scale-variable psychophysical model which assumes a number *n* is represented with standard deviation *wn*. This model predicts binary accuracy judgements on specific numerical comparisons, which is the observed data for the model. In addition, the model includes parameters to capture variation in species, tasks and individuals via a simple mixed-effect component. Thus, the model jointly fits effects of predictors across species on *w*, as well as the influence of phylogenetic relatedness employing a mixed-effect component similar to previous phylogenetic approaches [124], to control for individuals, study, species and task. Task refers to experimental task (i.e. paradigm), while study indicates a specific research study. A graphical model for our analysis is shown in figure 1. This shows the relationships between the key variables, with plates (rectangles) illustrating arrays of variables. For example, lambda, a parameter that controls the strength of the phylogenetic component, determines the species effects and is constant across all species, while *x*, the predictor of interest, takes on a different value for each species and, along with the regression coefficients *β*_0_ and *β*_1_, determines the mean value *µ* of each Weber fraction. This model importantly allows us to quantify the effect of each predictor under tight controls for these other factors, as well as a principled likelihood that is based on a formalization of approximate number perception.

Our model differs from standard phylogenetic regressions in that the quantity we measure, Weber fraction, is not directly observed. It is a latent variable that predicts accuracy only via a particular psychophysical linking function. We have a fixed covariance matrix *V* based on one phylogenetic tree which indicates how correlated species should be based on their phylogeny. That matrix *V* is mixed with the identity matrix via lambda (e.g. *M* = *λ* × *V* + (1 − *λ*) × identity) to create the inferred covariance matrix *M* among species. If *λ =* 0, each species effect is independent (sampled from standard normal), while if *λ =* 1, each species' adjustment to the overall Weber fraction is determined by the phylogeny's predicted covariance. In the model, lambda is fitted simultaneously with all other parameters (only the matrix *V* is fixed). The species-level adjustments to log Weber fraction are sampled from a normal with covariance *M*. In the model, these species effects are added to the grand overall (log) Weber fraction, effects for each task, individual and study. And then the (log) Weber fraction thus derived is used to predict the correct/incorrect outcomes on each number comparison using a standard psychophysical model. The only data we observe are *V* and the accuracies, while the effective Weber fraction for each trial is latent, as is the species effects on this Weber fraction.

We implemented the model in the Stan programming language (Stan v. 2.19.1, using the R interface rstan [125]), which employs Hamiltonian Monte Carlo, specifically the no-U-turn sampling (NUTS) algorithm [126]. Rhat values, meaning the agreement of multiple chains, were used to assess convergence; we found all Rhat values < 1.05 (indicating that between-chain variance was similar to within-chain variance).

## 3. Results

**Figure 2.**
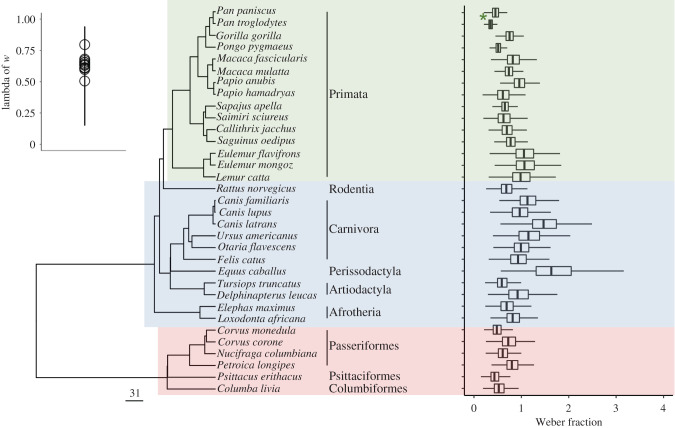
Phylogenetic tree of the species included in these analyses and the overall Weber fraction for each species (posterior quantiles shown, generated via a model with no predictors and only species, study, subject and task effects). These Weber fractions are based on quantity discrimination performance data from 672 individual animals across 33 species. Scale bar represents 31 Myr. Primates are highlighted in green, nonprimate mammals in blue and birds in red. Human Weber fraction (from [127]) indicated with green asterisk for comparison. The inset shows the lambda of *w* across all predictors with mean minimum and maximum of posterior distributions for lambda shown. (Online version in colour.)

Figure 2 shows a phylogenetic tree of the species included in these analyses and overall Weber fraction, with uncertainty represented, for each species. Larger Weber fractions reflect worse performance—a Weber fraction of 0.2 would mean an animal can reliably discriminate quantities as fine as 10 versus 12 whereas a Weber fraction of 0.5 affords reliable discrimination only to 10 versus 15. The ranges in Weber fractions in each of the three groups broadly agree with previous studies in primates [7,128–130], other mammals and birds [131]. Birds (in red) have Weber fractions that are lower and narrower posterior distributions than those of nonprimate mammals (blue) and some primates (green). Among nonprimate mammals, the horse and the coyote show highest estimated Weber fractions with the widest posterior distributions. Among primates, smaller Weber fractions with narrow posterior distributions are found for some Central and South American primates, and some Afro-Eurasian primates, including most apes. The human Weber fraction [127] is shown with a green asterisk for comparison with the other species. The inset in figure 2 shows lambda of *w* across all predictors (controlled), indicating the existence of a phylogenetic correlation for *w* across brain, socioecological and cognitive predictors, with uncertainty. Lambda indicates whether the species-level adjustments to Weber fraction are correlated in the way the phylogeny would predict or not; therefore, the inset in figure 2 provides evidence that *λ* > 0, which means that species are not independent. Subsequent results reported below take into account this uncertainty in phylogenetic correlation for *w*.

**Figure 3.**
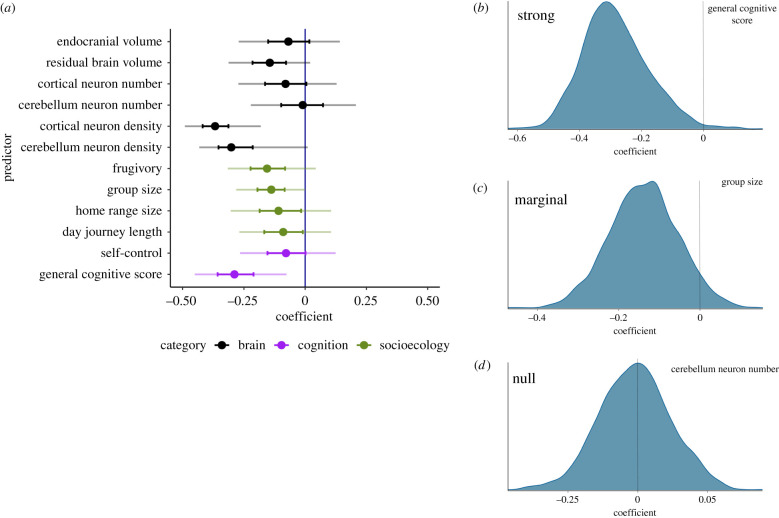
(*a*) Posterior distribution for the coefficient for each predictor from a model run with 33 species. 95% credible interval represents the central 95% of the posterior distribution (there is a 95% probability that the parameter lies within the 95% credible interval), while the 50% credible interval represents the central 50% of the posterior distribution. A parameter with no effect would be centred at 0 (as we found for cerebellum neuron number) with wide symmetrical error bars. To further characterize the distribution of posterior samples, posterior kernel density plots of a (*b*) strong effect (general cognitive score), (*c*) marginal effect (group size) and (*d*) null effect (cerebellar neuron number) are presented. Kernel density plots show posterior samples with chains merged.

In figure 3, the intervals illustrate the posterior distribution for the coefficient for each predictor. Posterior distributions in Bayesian analysis quantify what we should believe about the true value of the parameters, i.e. what we normatively should believe about the size of each effect. Specifically, figure 3 shows posterior uncertainty intervals that indicate the means that give the best estimate with a spread that shows how confident to be in that estimate. In our model, parameters are chosen so that their value quantifies a theoretically driven hypothesis, such as the likely influence of a predictor on numerical acuity. We plot posterior quantiles, which provide the range of likely true values for the parameters (e.g. the 50% quantiles show the most central half of probability, or belief, in the parameter's value). Bayesian analysis does not typically ask whether variables are exactly equal to zero—as in a null hypothesis test, or standard frequentist regression—because that has zero probability of being true in the world. Instead, these ranges illustrate the model's best guess (value) and confidence (range) in each effect. In figure 3, from a model with 33 species, the posterior distributions demonstrate that *w* decreases (quantity discrimination improves) with increased cortical neuron density, cerebellar neuron density and general cognitive score; weaker effects are also shown for RBV, frugivory and group size. A parameter with no effect would be centred at 0 (as cerebellum neuron number is) with wide symmetrical error bars. These findings indicate that a subset of neural and general cognitive variables are related to the evolution of quantitative sensitivity among species. A model with only primate species (*n* = 15) showed some effects, including cerebellar neuron density and self-control, were weakened or removed; cerebellar neuron density shifted to no effect (centred at zero) (electronic supplementary material, figure S1). Two effects were strengthened when only primates were included in the model: primate species *w* decreases (quantity discrimination improves) with increased RBV and increased cerebellum neuron number (electronic supplementary material, figure S1).

**Figure 4.**
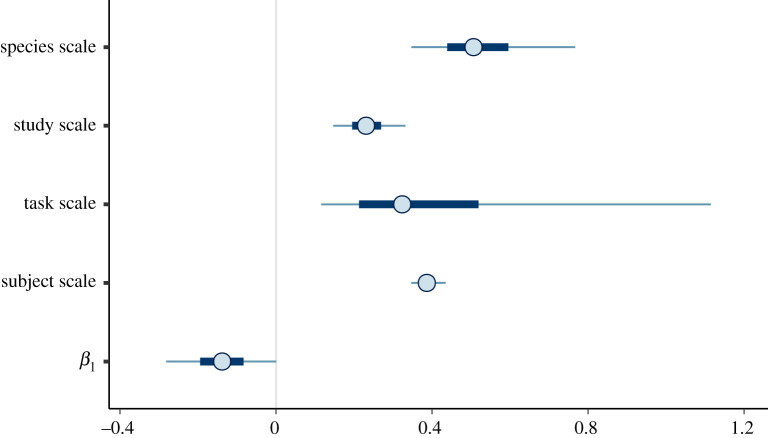
Posterior credible intervals (circles, posterior means; thick segments, 50% intervals; thin lines, 90% intervals) for the scale parameters and one predictor parameter chosen as an example (group size) run with 33 species. Each of these scales is a factor that multiplies the predictor parameter (e.g. for species, study, task, subject), and *β*_1_ multiplies the predictor (e.g. group size), making them comparable in indicating how much change in log *w* there is for each change in the predictor.

Figure 4 shows the posterior credible intervals on the scale parameters for species, subject, study and task, as well as the posterior credible interval of one of the predictors (group size). Figure 4 indicates that species and individual subject contribute to performance. Task, with wide uncertainty, also contributes to performance.

**Figure 5.**
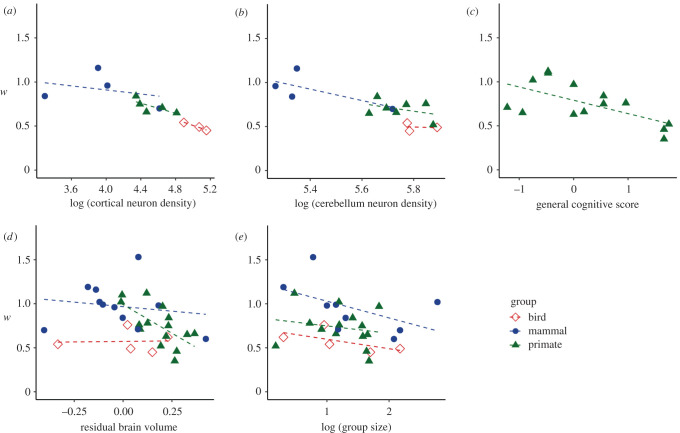
Scatterplots show species *w* (generated via a model with no predictors and only species, study, subject and task effects) by (*a*) cortical neuron density, (*b*) cerebellum neuron density, (*c*) general cognitive score, (*d*) residual brain volume and (*e*) group size. Mammals are blue circles, primates are green triangles and birds are red diamonds. (Online version in colour.)

Scatterplots shown in figure 5 allow us to visualize each predictor by effectively estimating *w* independently and examining its relationship with key predictors, by taxonomic group. The species *w* values in figure 5 were generated via a model with no predictors and only species, study, subject and task effects (i.e. the same model as for figure 1). The cortical neuron density (figure 5*a*) effect is driven by primates and birds, while the cerebellum neuron density effect is driven by nonprimate mammals (figure 5*b*). Better (lower) *w* was related to better general cognitive score in primates (figure 5*c*). For weaker effects, the RBV effect is driven by primates (figure 5*d*) and the group size effect is driven by all three taxonomic groups (figure 5*e*). The largest group size (with a relatively high *w*) belongs to the South American sea lion.

We acknowledge that *w* for different tasks may be related to predictors differently: for example, self-control may specifically be related to the sequential task *w*. In the sequential numerical cognition task, animals wait while a set of items is baited one-by-one over time, and this is a general task demand on attention and response control (e.g. [129]). Testing these predictions about task differences is outside the scope of our model because by assumption in the model, each task scales the value of *w* the same across all other predictors. Future work with different modelling approaches should address these questions as different quantitative tasks likely have unique relations to some predictors—particularly if the task requirements have consistent effects on performance among individuals of a species.

## 4. Discussion

The question of how strongly phylogeny predicts variation in cognition across species has a contentious intellectual history. For nearly a century, many psychologists considered evolution to be irrelevant to the study of learning because the behaviours of all species were thought to obey similar laws [132,133]. Ultimately, however, it was shown that the laws of learning often depend on the biological preparedness of the organism [134]. Comparative psychologists have since made inferences about species-specific biological influences and possible evolutionary relations among cognitive faculties but often relied on few species for comparative study [46]. By contrast, modern phylogenetic modelling methods applied across hundreds of individuals from dozens of species provide a new empirical test of phylogenetic constraints on cognition [48]. This is the first study to measure the origins of quantitative cognition with these methods. We found contributions of phylogeny to quantity discrimination performance across taxa, indicating evolutionary constraints on quantitative cognition. Additionally, a subset of neuronal and cognitive variables predicted species' quantitative sensitivity—the strongest predictors were neuron density and general cognitive ability. The results indicate that when selecting an animal from the world at random, we can roughly predict its Weber fraction by knowing its species.

An individual's Weber fraction was related to its species-typical cortical neuron density. Individuals from species with higher cortical neuron density had more precise Weber fractions. Thus, one constraint on an individual's quantitative cognition is the biological capacity for information processing in their brain, as determined genetically and developmentally for each species [74–81]. Additionally, quantitative precision was related to neuron density in the cerebellum, a brain structure that has been overlooked in studies of vertebrate brain evolution and cognition [89–91]. We found that density of neurons was a more accurate proxy for quantitative ability than brain volume when comparing species across taxa. Caveats to the interpretation of the neuron density findings include (i) the number of species with cortical and cerebellar neuron density values is lower than for neuronal number, and (ii) the relationship between neuron number and neuron density differs across animal groups. An increase in number of neurons in a primate brain structure does *not* mean larger neurons (and lower density), whereas in most nonprimate mammals more neurons means larger neurons and lower density [75,115]. Our study is a rare demonstration of a relation between neuron number or neuron density and a particular cognitive ability. Previous within-species comparisons showed that neuron number in multiple brain regions did not predict performance on a battery of behavioural tasks in mice [135], and though raccoons who performed best on a puzzle box task had more cells in their hippocampus than lower performing individuals, this difference may have been driven by glial cells [136]. However, cross-species comparisons in primates and birds suggest that neuron number has more behavioural explanatory power than cranial capacity, based on the correlation between cortical or pallial neuron number and performance on a self-control task [71]. Our cross-species finding from birds and mammals implies that quantitative sensitivity is yoked to species-specific developmental programmes for neuronal density; therefore, some species are well-equipped to develop precise quantitative sensitivity whereas others may be unable to do so.

Our finding that primate species' quantitative sensitivity improved with their domain-general cognition score indicates that general cognitive functions, perhaps in tandem with specialized quantitative functions, impacted the evolution of quantitative precision across species. In the original domain-general cognition study [120], primates’ performance on tasks like object discrimination, reversal learning, sorting, oddity learning and delayed response reliably approximated general cognitive ability at the genus level in a Bayesian latent variable analysis. This ‘general cognitive ability’ variable could represent a constellation of cognitive capacities that distinguishes species, or it could represent attention or memory functions that affect animals' overall task performance [105]. The current study expands the suite of tasks that relate to general cognitive ability to include quantitative sensitivity, which provides further support for the hypothesis that some species perform better than others across tasks independently of task content. These conclusions about general cognitive factors are limited to primates and it is an open question whether these patterns generalize to other mammals and birds. However, it is remarkable that these general cognitive scores may mediate differences in quantitative cognition between species as closely related as, for example, an olive baboon and a capuchin monkey.

It is important to highlight that these anatomical, ecological and cognitive factors are likely interrelated [51,137,138], meaning that some factors may play a role in mediating others. For instance, the influence of foraging pressures may be difficult to separate independently from group size. However, in general, separating out the independent *causal* contributions of correlated components is difficult, even more so in analyses like these where all variables were not available for all species. Our analysis, therefore, focused on evaluating each factor in a simplified framework where each is tested independently. Additional data and methods are needed to disentangle how social, ecological and brain selection pressures are intertwined in affecting cognition [139] and for exploration of mosaic evolution of cognition, in which social and/or ecological variables shape particular domains differently [100,140]. Fully formalized theories of how such causal factors jointly interact may permit computational modelling of further relationships in the future.

Our novel analysis shows that biological features of a species’ evolutionary history likely modulate the development of individuals' numerical cognition, a crucial finding that emphasizes the importance of phylogenetic constraints on cognition. Natural selection has biologically prepared some species to develop high neuronal densities and general cognitive capacities that yield precise quantitative representations. These data begin to reveal the evolutionary pressures that shaped numerical cognition across species and bring us closer to understanding the evolutionary precursors that sparked human mathematical cognition.

## Data accessibility

Data files, Stan models and R scripts used in these analyses are available via the Open Science Framework (OSF) at: https://osf.io/j9sv8/ (doi:10.17605/OSF.IO/J9SV8) and via Carnegie Mellon University's repository KiltHub, doi:10.1184/R1/17144432.
